# Parental willingness to vaccinate male children against human papillomavirus in the Makkah region of Saudi Arabia: a study based on the health belief model

**DOI:** 10.3389/fpubh.2025.1558221

**Published:** 2025-06-27

**Authors:** Fahad T. Alsulami

**Affiliations:** Clinical Pharmacy Department, College of Pharmacy, Taif University, Taif, Saudi Arabia

**Keywords:** HPV, HPV vaccine, HBM, knowledge, male children, parent

## Abstract

**Objective:**

This study aimed to evaluate parental willingness to vaccinate their male children against human papillomavirus in the Makkah region of Saudi Arabia, and to explore the influence of Health Belief Model constructs on this willingness.

**Methods:**

A cross-sectional survey was conducted among parents aged 18 years and older residing in the Makkah region of Saudi Arabia, each with at least one male child. Participants were recruited using non-probability convenience sampling. Data were collected through a self-administered online questionnaire assessing HPV-related knowledge, willingness to vaccinate, and Health Belief Model constructs. Descriptive statistics, chi-square tests, and independent samples t-tests were used to assess group differences. Multivariable logistic regression analysis was conducted to identify factors associated with willingness to vaccinate, adjusting for sociodemographic variables.

**Results:**

A total of 604 parents participated, with approximately 40 percent expressing high willingness to vaccinate their male children. Parents who demonstrated greater willingness had better knowledge about human papillomavirus and its vaccine, perceived higher susceptibility of their children to the virus, viewed the infection as more severe, recognized greater benefits of vaccination, and identified more cues to take action compared to those with lower willingness. Multivariable logistic regression analyses, adjusted for relevant sociodemographic variables, indicated that perceived susceptibility (odds ratio = 2.85; *p* < 0.001), perceived benefits (odds ratio = 1.88; *p* = 0.020), and cues to action (odds ratio = 2.92; *p* < 0.001) were significantly associated with willingness to vaccinate.

**Conclusion:**

The study highlights that parental willingness to vaccinate male children against human papillomavirus in the Makkah region is significantly influenced by perceived susceptibility, perceived benefits of the vaccine, and cues to action. Interventions aimed at increasing awareness of the risks associated with human papillomavirus and the benefits of vaccination, as well as strengthening actionable prompts, may enhance vaccination uptake.

## 1 Introduction

Human papillomavirus (HPV) is a common viral infection, primarily transmitted through sexual contact. It is recognized as the most prevalent sexually transmitted infection globally. Over 200 genotypes of HPV have been identified, of which approximately 14 are considered high-risk or oncogenic types (1). HPV affects approximately 31% of men globally (2). Most individuals infected with HPV show no symptoms, leading to widespread undiagnosed cases and, consequently, an increased potential for the infection to spread (3). The international data support the role of HPV in oropharyngeal, anal, and penile cancers in men. Although many cases are asymptomatic and go undiagnosed (3), the long-term consequences of persistent infection with high-risk HPV types are significant. Globally, more than 70,000 cancer cases in men were attributed to HPV infection in 2019, affecting sites such as the oropharynx, anus, and penis (4). In the United States, over 21,000 cases of HPV-related cancer are observed among men annually, with oropharyngeal cancer being the most prevalent form of HPV-associated cancer among males (5). Moreover, HPV accounts for more than 85% of male penile cancer cases and 60% of anal cancer cases. HPV leads to 900 cases of penile cancer and 2,100 cases of anal cancer among men every year in the United States (6).

In recent years, increasing attention has been directed toward HPV-related disease burden among males, highlighting the importance of including boys in vaccination programs to reduce the overall prevalence of HPV-associated cancers (7). Vaccination is the most effective measure to prevent HPV infection and its associated diseases. Currently available HPV vaccines, including Gardasil®, Gardasil-9®, and Cervarix®, demonstrate high efficacy in preventing oncogenic strains of HPV (8, 9). Initially, the HPV vaccine was administered primarily to girls between the ages of 11 and 12, with catch-up vaccination provided to females aged 13 to 26 years (10). In 2018, the United States FDA expanded the coverage of HPV vaccination to include both male and female up to 45 years of age (11).

In Saudi Arabia, the Saudi Food and Drug Authority (SFDA) approved the use of HPV vaccines in 2010, specifically Cervarix® and Gardasil®, with initial implementation focused on females aged 11–26 years (12). Subsequently, in 2018, the HPV vaccine was formally integrated into the Saudi National Immunization Schedule for routine administration to females, whereas it remains an optional intervention for males (13, 14). Currently, HPV vaccines are available free of charge at public hospitals and primary healthcare centers. In addition, HPV vaccination may be administered during routine consultations in pediatric and family medicine clinics. Importantly, both male and female individuals in Saudi Arabia are eligible to receive the vaccine upon request.

Optimal protection from HPV is achieved when vaccination occurs at a younger age, typically between 11 and 12 years, before potential exposure to the virus (15). As a result, the successful implementation of vaccination relies heavily on parents accepting the vaccine, especially during years when parental guidance is crucial (16). Therefore, the initial effort to encourage male HPV vaccination should focus on engaging parents. Several factors influence parents’ decisions to vaccinate their male children with the HPV vaccine. Parents who perceived their male children to be vulnerable to HPV infection were more willing to have them receive the vaccine (17, 18). Conversely, parents who perceived HPV as a less serious infection in males were less likely to have their male children vaccinated (19). Additionally, parents who are concerned about the safety of the HPV vaccine are less willing to have their male children vaccinated with it (20, 21). However, parents with greater knowledge and awareness of HPV and its vaccine demonstrate a greater willingness to vaccinate their male children against HPV (17).

The health belief model (HBM), a type of cognitive theory, serves as a theoretical framework for understanding how perceived susceptibility, perceived severity, perceived benefits, perceived barriers, and cues to action contribute to the formation of health-related behaviors (22). The HBM is widely employed as a conceptual framework in health behavior research, including immunization against transmitted infections, and serves to elucidate changes in health-related behaviors, offering guidance for the development of interventions (22, 23).

Despite the growing recognition of the importance of male HPV vaccination, there is a paucity of research in Saudi Arabia examining the cognitive and behavioral factors that influence parental decision-making. Existing studies have primarily focused on female vaccination, with limited exploration of male-focused interventions. Furthermore, few studies in the region have utilized theoretical models like the HBM to systematically investigate these factors. Parents with more knowledge about HPV and related vaccines are more willing to vaccinate their daughters with the HPV vaccine (24). On the other hand, parents who perceive a low risk of their children acquiring HPV are less likely to vaccinate them (25). Doubt about the effectiveness of the HPV vaccine and fear of its side effects are barriers to vaccinating children among parents in Saudi Arabia (26).

Given the evolving global recommendations advocating gender-neutral HPV vaccination and the optional nature of male vaccination in Saudi Arabia, it is essential to understand parental attitudes and perceptions to inform public health strategies and potential policy expansion (27). Early assessment of these factors is especially important in regions like Makkah, where public health education can play a pivotal role in shaping future vaccination behavior.

Therefore, the objective of this study is to assess parental willingness to vaccinate their male children against HPV in the Makkah region of Saudi Arabia, using the HBM as a conceptual framework. By focusing on an understudied demographic and applying a validated theoretical model, this research aims to contribute novel insights into the sociocognitive determinants of HPV vaccine acceptance in a culturally specific context, thereby informing future public health initiatives and policy development. Furthermore, by emphasizing male children, an often underrepresented group in HPV vaccination research, and adopting a rigorously validated theoretical approach, the study offers novel insights into the psychological and sociocultural dimensions that shape vaccine acceptance. These findings are intended to contribute to the limited body of evidence on male HPV vaccination in Saudi Arabia and to support the development of culturally sensitive health education initiatives and policy measures. Ultimately, the research aims to inform targeted interventions designed to enhance HPV vaccine uptake and mitigate the long-term public health burden of HPV-related diseases among both genders in the region.

## 2 Methods

This study employed a cross-sectional survey using convenience sampling, a nonprobability sampling technique, to recruit parents in the Makkah region of Saudi Arabia. Data collection was carried out through an online self-administered questionnaire survey using Google Forms. The survey link was distributed to parents via Telegram and WhatsApp groups. Additionally, the author requested that college students aid in distributing the survey link. To be eligible, participants had to be at least 18 years old, have at least one male child, be capable of providing informed consent, and have resided in the Makkah region of Saudi Arabia for a minimum of 6 months prior to participation.

The required sample size was calculated using the formula for estimating proportions in a large population: *n* = *Z*^2^ × *P*(1–*P*) / *d*^2^, where *Z* is the *Z*-score for a 98% confidence level (2.33), *P* is the estimated prevalence (0.5), and d is the margin of error (0.05). Based on the total population of 8,325,304 in the Makkah region (28), and accounting for a 10% non-response rate, the final minimum sample size was calculated to be 596. A total of 604 eligible parents participated in the study, which meets and slightly exceeds the required sample size, enhancing the study’s statistical power and generalizability.

### 2.1 Data collection

The research survey comprised a set of validated instruments were adapted from the litreture (29, 30). It included a sociodemographic instrument, an instrument measuring parents’ willingness to vaccinate their male children against HPV, an instrument assessing parents’ knowledge about HPV and the related vaccine, and five constructs of the HBM instrument (perceived susceptibility, perceived severity, perceived benefits, perceived barriers, and cues to action). These instruments were derived from relevant literature and reviewed by two proficient academic pharmacists. Changes were implemented according to the feedback provided by the reviewers. The survey was subjected to evaluation for face and content validity checks through a pilot test involving 10 parents, and their responses were excluded from the final analysis. The survey items were designed to be simple and delivered in both English and Arabic to avoid misunderstandings. Additionally, the reliability of the knowledge instruments and the five constructs of the HBM were assessed via Cronbach’s alpha test.

#### 2.1.1 The willingness of parents to vaccinate their male children against HPV

Parents’ willingness to vaccinate their male children against HPV was measured via a single item (i.e., Are you willing to get your son(s) vaccinated against HPV?). A 5-point Likert scale (strongly disagree = 1, strongly agree = 5) was employed to capture parents’ responses. An average score between 1 and 5 was computed, where a higher score denoted a greater willingness to vaccinate. The overall mean score was subsequently used as the cutoff point to classify parents into two categories: those with a low willingness to vaccinate their male children against HPV (coded as 0) and those with a high willingness to vaccinate their male children against HPV (coded as 1), consistent with previous studies (31, 32).

#### 2.1.2 Knowledge about HPV and the HPV vaccine

Parents were asked a series of questions about HPV and the HPV vaccine via a 19-item scale adapted from literature (e.g., “Human papillomavirus (HPV) infection always has visible signs or symptoms,” “Men cannot be infected with Human papillomavirus (HPV),” and “The Human papillomavirus (HPV) vaccines offer protection against most HPV-related cancers”) (29). Parents responded with True, False, or Do not Know. A score of one was given for each correct response, whereas incorrect or “Do not Know” responses received a score of zero. The overall knowledge score ranged from 0 to 19, with higher scores reflecting better knowledge of HPV and the vaccine. The knowledge scale showed strong internal consistency, as evidenced by a Cronbach’s alpha of 0.88.

#### 2.1.3 Health belief model

The HBM instrument consists of five components drawn from the literature, including perceived susceptibility, severity, benefits, barriers, and cues to action (30). Three items were utilized to assess perceived susceptibility (e.g., “Without the HPV vaccine, my male children would be at risk of contracting HPV later in life”). Similarly, the perceived severity of HPV was assessed via three items (e.g., “It would be serious if my male children contracted HPV later in life”). The perceived benefits of vaccinating their male children with the HPV vaccine were evaluated with five items (e.g., “The HPV vaccine is effective in preventing HPV”). Additionally, perceived barriers to vaccinating their male children with the HPV vaccine were measured with three items (e.g., “The HPV vaccine is unsafe”). Finally, cues to action were evaluated via four items (e.g., “Other parents in my community and my friends would get their male children vaccinated against HPV”). The parents’ responses were collected via a 5-point Likert scale. For each HBM construct, an average score ranging between 1 and 5 was computed, where a higher score indicated that parents perceived a high susceptibility of their male children to contracting HPV, a high severity of HPV infection, high benefits of vaccinating their male children with the HPV vaccine, high barriers to vaccinating their male children with the HPV vaccine, or more cues to vaccinating their male children with the HPV vaccine. The internal consistency of each HBM construct scale demonstrated reliability, as measured by Cronbach’s alpha, with values ranging from 0.85 to 0.94.

### 2.2 Data analysis

This study utilized a descriptive method to characterize and summarize the dataset. An independent samples t test was conducted to identify differences in knowledge about HPV and the HPV vaccine, as well as variations in perceptions of susceptibility, severity, benefits, barriers, and cues to action between parents with high and low willingness to vaccinate their male children with the HPV vaccine. The chi-square test was employed to identify variations in parents’ willingness to vaccinate their male children on the basis of different sociodemographic characteristics. Additionally, an entry binary logistic regression analysis was conducted to examine the association between independent variables and the outcome variable (parental willingness to vaccinate male children against HPV). Independent variables included sociodemographic factors, HPV-related knowledge, and HBM constructs. Variables demonstrating a *p*-value < 0.25 in the initial binary logistic regression were considered candidates for inclusion in the subsequent multivariable logistic regression mode. Adjusted odds ratios (AORs) with 95% confidence intervals (CIs) were reported to measure the strength and precision of associations in the multivariable model. Statistical significance was determined using a two-sided *p*-value threshold of < 0.05. The Nagelkerke R^2^ statistic was also reported to indicate the proportion of variance in the outcome variable explained by the model. All analyses were conducted via the IBM® Statistical Package for the Social Sciences (SPSS), version 29.0.

## 3 Results

A total of 930 parents initially submitted the survey the online survey during the data collection period. Of the 930 parents, 326 responses were excluded from the final analysis. Specifically, 42 submissions were removed due to incomplete data, and 284 were excluded because the respondents did not have male children, which was a primary inclusion criterion. Consequently, 604 parents met all eligibility criteria and were included in the final analysis, representing an inclusion rate of 64.9%. The mean age of participants was 43 years (SD = 9.90). Approximately 75% of them were female, and approximately 56% of the parents had two to four male children. Additionally, approximately 62% had a bachelor’s degree. Approximately 60% of the parents were employed (Table 1).

**Table 1:** Sociodemographic characteristics of parents participating in the study [*N* = 604].

Sociodemographic characteristics	Frequency	Percent (%)
Gender		
Male	150	24.8%
Female	454	75.2%
Education		
High school or less	135	22.4%
Diploma degree	43	7.1%
Bachelor’s degree	376	62.3%
Higher education degree	50	8.3%
Number of male children		
One male child	207	34.3%
2 to 4 male children	340	56.3%
5 male children or more	57	9.4%
Employment status		
Employed	361	59.8%
Not employed	243	40.2%

### 3.1 Parents’ willingness to vaccinate their male children with the HPV vaccine

Regarding parental willingness to vaccinate their male children against the HPV vaccine, the mean score on the willingness scale was 3.18 out of 5. As specified in the Methods section, this mean value was employed as a threshold to dichotomize the responses. Participants with scores exceeding the mean were classified as exhibiting a high level of willingness. Based on this criterion, approximately 40% of respondents were identified as having a high willingness to vaccinate their male children against HPV.

A chi-square analysis was conducted to examine significant variances in parental willingness rates across various sociodemographic characteristics. Approximately 47% of male parents and 37% of female parents exhibited high willingness to vaccinate their male children with the HPV vaccine (*p* value 0.028). Additionally, high willingness rates were reported to be 54, 43, and 35% for parents who had five or more male children, one male child, and two to four male children, respectively (*p* value = 0.012) (Table 2).

**Table 2:** Willingness of parents to vaccinate their male children with the HPV vaccine by sociodemographic characteristics [*N* = 604].

Sociodemographic Characteristics	Parents with high willingness to vaccinate their male children against HPV (*n* = 240)	Parents with low willingness to vaccinate their male children against HPV (*n* = 364)	*p* value
Gender			**0.028**
Male	47.3%	52.7%	
Female	37.2%	62.8%	
Education			0.181
High school or less	33.3%	66.7%	
Diploma degree	32.6%	67.4%	
Bachelor’s degree	42.0%	58.0%	
Higher education degree	46.0%	54.0%	
Number of male children			**0.012**
One male child	43.0%	57.0%	
2 to 4 male children	35.3%	64.7%	
5 male children or more	54.4%	45.6%	
Employment status			0.346
Employed	41.3%	58.7%	
Not employed	37.4%	62.6%	

Bold means *p*-vlaue < 0.05.

### 3.2 Parents’ knowledge levels of HPV and the HPV vaccine and HBM constructs

Parents had very low knowledge levels about HPV and the related vaccine; the overall mean knowledge score was 5.97 out of 19. Furthermore, parents perceived a moderate susceptibility of their male children to acquire HPV (*mean* = 3.02), a moderate severity of HPV (*mean* = 3.35), and moderate benefits of vaccinating their male children with the HPV vaccine (*mean* = 3.36). Additionally, parents perceived low to moderate barriers (*mean* = 2.92) and moderate cues (*mean* = 3.14) to vaccinating their male children with the HPV vaccine.

An independent samples t test was used to evaluate the differences in average knowledge and the five HBM constructs among parents on the basis of their willingness to vaccinate their male children with the HPV vaccine. The findings showed that parents who were more willing to vaccinate their male children had significantly greater knowledge about HPV and the HPV vaccine than those who were less willing (*p* value < 0.001). Moreover, parents with high willingness to vaccinate their male children with the HPV vaccine perceived greater susceptibility of their male children to HPV, perceived greater severity of the disease, and perceived greater benefits from the HPV vaccination than did those with low willingness (*p* value < 0.001). Additionally, parents with high willingness to vaccinate their male children with the HPV vaccine perceived more cues to action to vaccinate their male children than did those with low willingness (*p* value < 0.001). However, there was no significant difference in perceived barriers to vaccination between the two groups (*p* value = 0.146) (Table 3).

**Table 3:** Mean scores of parental knowledge about HPV and the HPV vaccine, and health belief model constructs by willingness to vaccinate [mean (SD), *N* = 604].

Parents’ willingness statues	HPV and the HPV vaccine knowledge Mean (SD)	Perceived susceptibility Mean (SD)	Perceived severity Mean (SD)	Perceived benefits Mean (SD)	Perceived barriers Mean (SD)	Cues to action Mean (SD)
Parents with high willingness to vaccinate their male children against HPV	7.49 (4.74)	3.52 (0.79)	3.72 (0.83)	3.76 (0.62)	2.86 (0.88)	3.54 (0.65)
Parents with low willingness to vaccinate their male children against HPV	4.97 (4.65)	2.69 (0.72)	3.10 (0.85)	3.09 (0.68)	2.95 (0.65)	2.87 (0.60)
*p* value	**<0.001**	**<0.001**	**<0.001**	**<0.001**	0.146	**<0.001**

Bold means *p*-vlaue < 0.05.

### 3.3 Predictors affecting parents’ willingness to vaccinate their male children with the HPV vaccine

A multivariable logistic regression analysis was conducted to assess the influence of various factors, including sex, age, education level, number of male children, knowledge levels of HPV and the HPV vaccine, along with five HBM constructs, on parental willingness to vaccinate their male children against HPV. The results of the analysis revealed significant findings (χ^2^ = 239.192, *p* value < 0.001), indicating that the model accounted for approximately 44% of the variance in parental willingness to vaccinate their male children against HPV (Nagelkerke *R^2^* = 0.442). Notably, having five or more male children was found to be significantly associated with an increased likelihood of parents being more willing to vaccinate their male children against HPV (*OR* = 2.36; *p* value = 0.027).

Moreover, among the HBM constructs, only three were found to have significant associations with parental willingness to vaccinate their male children against HPV. Parents who perceived that their male children were highly susceptible to HPV infection were more inclined to express a high willingness to vaccinate them against HPV (*OR* = 2.85; *p* value < 0.001). Similarly, those who perceived high benefits from the HPV vaccine were more likely to express a high willingness to vaccinate their male children (*OR* = 1.88; *p* value = 0.020). Finally, parents who perceived more cues promoting the vaccination of their male children against HPV were also more likely to express a high willingness to vaccinate their male children (OR = 2.92; *p* value < 0.001) (Table 4).

**Table 4:** Multivariable logistic regression analysis of factors associated with parental willingness to vaccinate male children with the HPV vaccine [*N* = 604].

Predictor variables	Coefficient	SE	Wald	AOR	Lower CI (95%)	Upper CI (95%)	*p* value
Age	0.006	0.012	0.225	1.006	0.983	1.029	0.635
Gender							
Male	Reference						
Female	−0.378	0.264	2.045	0.685	0.408	1.150	0.153
Education							
High school or less	Reference						
Diploma degree	−0.344	0.484	0.503	0.709	0.274	1.833	0.478
Bachelor’s degree	0.415	0.263	2.504	1.515	0.906	2.535	0.114
Higher education degree	0.346	0.437	0.629	1.414	0.601	3.326	0.428
Number of male children							
One male child	Reference						
2 to 4 male children	−0.255	0.234	1.186	0.775	0.490	1.226	0.276
5 male children or more	0.861	0.390	4.871	2.365	1.101	5.079	**0.027**
Knowledge about HPV and the HPV vaccine	0.036	0.027	1.739	1.036	0.983	1.093	0.187
HBM constructs							
Perceived susceptibility	1.048	0.193	29.525	2.852	1.954	4.163	**<0.001**
Perceived severity	−0.108	0.168	0.413	0.898	0.646	1.247	0.520
Perceived benefits	0.633	0.271	5.448	1.882	1.107	3.202	**0.020**
Perceived barriers	0.046	0.180	0.066	1.047	0.736	1.490	0.798
Cues to action	1.072	0.241	19.713	2.922	1.820	4.690	**<0.001**

Bold means *p*-vlaue < 0.05.

## 4 Discussion

HPV is the most prevalent sexually transmitted infection among men, with more than 30% of men globally being infected with it (2). In 2019, over 70,000 HPV-related cancer cases were diagnosed among men globally (4). Vaccination against HPV is the most effective way to protect both men and women from the majority of HPV-related cancers. Since the introduction of HPV vaccines, few studies have assessed parents’ willingness to vaccinate their female children with the HPV vaccine in Saudi Arabia (24, 33). However, no known study has assessed factors associated with parents’ willingness to vaccinate their male children with the HPV vaccine in Saudi Arabia via the HBM. This study is one of the first studies that aimed to examine the effects of HBM constructs on parents’ willingness to vaccinate their male children with the HPV vaccine in the Makkah region of Saudi Arabia.

The findings of this study indicate that fewer than half of parents were willing to vaccinate their male children with the HPV vaccine. Although previous studies in Saudi Arabia have primarily assessed parental willingness to vaccinate female children, they similarly reported low acceptance rates (24, 33). One possible explanation for this finding could be that parents in Saudi Arabia have limited awareness and knowledge of HPV and its vaccine (25). Studies have shown that this lack of knowledge is associated with a decreased willingness among parents to vaccinate their children against HPV (34, 35). Therefore, there is a clear need for increased efforts to increase awareness and knowledge levels about HPV and its vaccine among parents in Saudi Arabia, with the aim of increasing HPV vaccination rates.

In addition, the findings of the study indicated that male parents had higher odds of being willing to vaccinate their male children than female parents did, which is consistent with other studies conducted in Saudi Arabia and Ethiopia, where male parents showed higher willingness rates to vaccinate their children with the HPV vaccine than female parents did (33, 36). This might be explained by fathers having higher knowledge levels than mothers do about HPV and its vaccine (33). These findings emphasize the necessity of enhancing mothers’ knowledge and awareness of HPV and its vaccine.

In addition, this study revealed that parents with two to four male children had higher odds of being willing to vaccinate their male children with the HPV vaccine. This could be attributed to the possibility that parents who have a greater number of children are more inclined to have experienced positive effects of vaccinations on health. Furthermore, Alshehri and colleagues (33) reported that high rates of parents with four or more children had good knowledge about HPV and the HPV vaccine compared with those with three children or fewer in Saudi Arabia, but this difference was not statistically significant. However, further research is needed to explore the underlying reason for this trend and to develop targeted interventions to increase HPV vaccination uptake among all male children, regardless of their family composition. Understanding these factors is crucial for public health efforts aimed at reducing the incidence of HPV-related diseases and promoting overall population health.

The results of the present study indicate that parents had low knowledge about HPV and the HPV vaccine, which is consistent with findings from other studies conducted in Saudi Arabia (25, 37, 38). This lack of knowledge among parents may be attributed to insufficient educational initiatives about HPV and the HPV vaccine, particularly within primary healthcare facilities and public schools in Saudi Arabia. Therefore, there is a need for more HPV vaccination campaigns featuring educational materials tailored for parents in Saudi Arabia to increase knowledge and awareness. Additionally, the findings of this study revealed that parents who had higher odds of being willing to vaccinate their male children with the HPV vaccine presented greater knowledge about HPV and its vaccine than those who were less willing. This finding supports those of other studies conducted in Saudi Arabia, which showed that parents with greater knowledge about HPV and the HPV vaccine were more willing to vaccinate their daughters than were those with less knowledge (25, 33). Given the importance of parental knowledge in increasing HPV vaccination rates in Saudi Arabia, it is imperative to intensify efforts targeting parents to improve their understanding of HPV and its vaccine. This can be achieved through educational campaigns, community outreach programs, and ensuring that accurate information about HPV and the HPV vaccine is readily accessible to all parents. By enhancing parental understanding of the benefits and safety of HPV vaccination, we can potentially increase vaccine acceptance rates and ultimately reduce the burden of HPV-related diseases in Saudi Arabia.

This study indicates that only the perceived susceptibility, benefits, and cues to action of the HBM constructs were significantly associated with parents’ willingness to vaccinate their male children with the HPV vaccine after controlling for all other factors. Parents who perceived their male children as highly susceptible to contracting HPV infection had higher odds of being willing to vaccinate them, which is consistent with findings from other studies (17, 18). Therefore, healthcare providers and vaccination campaigns should focus on increasing parents’ awareness of HPV infection rates among males to increase HPV vaccination rates among male children in Saudi Arabia.

Additionally, parents who perceived high benefits of HPV vaccination had higher odds of being willing to vaccinate their male children against HPV, which aligns with findings from other studies (39, 40). The correlation between perceived benefits and willingness to vaccinate highlights the importance of effective communication strategies in conveying the advantages of HPV vaccination. Parents who understand the high benefits of the vaccine, such as reducing genital warts and the risk of HPV-related cancers, are more likely to vaccinate their male children with the HPV vaccine. Therefore, healthcare providers and the Ministry of Health should focus on disseminating information about the benefits of HPV vaccination through various channels, including educational campaigns, healthcare consultations, and community outreach programs.

Moreover, the current study revealed that parents who perceived more cues about vaccinating their male children with the HPV vaccine had higher odds of being willing to do so. This finding aligns with similar studies conducted in both the United States and Canada (41, 42). Furthermore, another study revealed that a lack of HPV vaccination recommendations was associated with not receiving the HPV vaccine among parents in Saudi Arabia (33). Nevertheless, a previous study conducted by Alnaeem and colleagues (26) revealed that only approximately 8% of parents received HPV vaccination recommendations from healthcare providers in Saudi Arabia. This suggests a significant gap in healthcare provider communication regarding the importance and necessity of HPV vaccination for children in Saudi Arabia. Addressing this disparity with enhanced educational programs and proactive recommendations could increase vaccination rates and alleviate the burden of HPV-related illnesses within the area.

## 5 Recommendation

Based on the study’s findings and guided by the Health Belief Model, several recommendations can be proposed to enhance parental willingness to vaccinate male children against HPV in the Makkah region of Saudi Arabia. First, public health authorities should implement culturally tailored educational interventions that address the substantial knowledge gap identified in the study. These programs should emphasize the risks associated with HPV infection in males and the benefits of vaccination, particularly in light of the observed association between perceived susceptibility and willingness to vaccinate. Second, the role of healthcare providers is paramount; the significant influence of cues to action suggests that structured, evidence-based communication by physicians, pediatricians, and pharmacists during routine consultations could serve as a powerful motivator for vaccine uptake. Third, engagement with religious and community leaders is essential to address culturally rooted misconceptions, such as concerns about the vaccine promoting promiscuity. Collaborating with these influential figures may facilitate the dissemination of health messages that align with Islamic values and promote HPV vaccination as a preventive health measure. Additionally, integrating HPV vaccination awareness into national immunization programs and school-based health initiatives can normalize the vaccine as part of standard adolescent care, thereby improving accessibility and acceptance. Finally, future research should further investigate the interplay between religious beliefs and vaccine attitudes in Saudi Arabia, as well as explore similarities with other culturally conservative populations. Such research can inform the development of context-specific strategies that enhance vaccine acceptance and address broader public health challenges related to vaccine hesitancy.

## 6 Limitations

There are several limitations that should be acknowledged. First, the utilization of convenience sampling restricts the generalizability of the findings to other populations. Second, the recruitment of parents solely from one region in Saudi Arabia restricts the applicability of the results to other regions within the country. Third, owing to the cross-sectional design employed, it is difficult to draw conclusions about causality. Fourth, reliance on self-report surveys introduces the possibility of recall bias, potentially skewing the strength of the observed correlations. Fifth, the lack of uniformity in the instrument based on the HBM utilized to evaluate parents’ behavior regarding vaccinating their male children with the HPV vaccine is another constraint of this study. Sixth, since the survey was self-administered and conducted online, it inherently required a minimum level of literacy and internet access, which may have excluded certain segments of the population. Saventh, the study analyzed knowledge about HPV and the HPV vaccine but did not examine levels of awareness of HPV and the HPV vaccine. This omission could result in an overestimation or underestimation of parental knowledge regarding HPV and its vaccine. Finally, this study did not evaluate the actual percentage of male children who had been vaccinated against HPV. Although this study concentrated on parental willingness to vaccinate, knowing the percentage of qualified sons who had already gotten the vaccination might have offered more insights on the elements driving willingness as well as those impacting real vaccine uptake. Future studies might fill this gap by including additional information on the vaccination status of eligible male children to better understand the link between willingness and actual vaccination behavior.

## 7 Conclusion

This study is among the few to explore how the constructs of the HBM influence parents’ willingness to vaccinate their male children against HPV in the Makkah region of Saudi Arabia. The findings reveal that only about 40% of parents were willing to vaccinate their sons, and there was a generally poor level of knowledge regarding HPV and the HPV vaccine. Among the HBM constructs, perceived susceptibility, perceived benefits, and cues to action were significantly associated with willingness to vaccinate. These results suggest that efforts to improve awareness of HPV risk and the benefits of vaccination, as well as targeted cues to action (such as recommendations by healthcare providers), may enhance vaccine uptake among this population.

## Data Availability

The raw data supporting the conclusions of this article will be made available by the authors, without undue reservation.
